# Exploring the immunological landscape of osteomyelitis through mendelian randomization analysis

**DOI:** 10.3389/fgene.2024.1362432

**Published:** 2024-04-08

**Authors:** Kehan Long, Ao Gong, Dou Yu, Sumiao Dong, Zhendong Ying, Lei Zhang

**Affiliations:** ^1^ School of Clinical Medicine, Shandong Second Medical University, Weifang, Shandong, China; ^2^ Second Clinical Medical College of Shandong University of Traditional Chinese Medicine, Jinan, Shandong, China; ^3^ School of Clinical Medicine, Shandong First Medical University, Jinan, Shandong, China; ^4^ The First Affiliated Hospital of Shandong First Medical University, Jinan, Shandong, China

**Keywords:** osteomyelitis, immune cell phenotype, mendelian randomization, genome-wide association study, causal relationship

## Abstract

**Background:**

Osteomyelitis is a severe bone marrow infection, whose pathogenesis is not yet fully understood. This study aims to explore the causal relationship between immune cell characteristics and osteomyelitis, hoping to provide new insights for the prevention and treatment of osteomyelitis.

**Methods:**

Based on two independent samples, this study employed a two-sample Mendelian randomization (MR) analysis to assess the causal relationship between 731 immune cell characteristics (divided into seven groups) and osteomyelitis. Genetic variants were used as proxies for risk factors to ensure that the selected instrumental variables meet the three key assumptions of MR analysis. Genome-Wide Association Studies (GWAS) data for immune characteristics were obtained from the public GWAS catalog, while data for osteomyelitis was sourced from the FinnGen.

**Results:**

At a significance level of 0.05, 21 immune phenotypes were identified as having a causal relationship with osteomyelitis development. In the B cell group, phenotypes such as Memory B cell % B cell (percentage of memory B cells within the total B cell population, % finger cell ratio), CD20^−^ %B cell (percentage of B cells that do not express the CD20 marker on their surface), and Memory B cell % lymphocyte showed a positive causal relationship with osteomyelitis, while Naive-mature B cell %B cell and IgD-CD38-absolute cell counts (AC) phenotypes showed a negative causal relationship. In addition, specific immune phenotypes in the conventional dendritic cells (cDCs) group, Myeloid cell group, TBNK (T cells, B cells, natural killer cells) cell group, T cell maturation stage, and Treg cell group also showed significant associations with osteomyelitis. Through reverse MR analysis, it was found that osteomyelitis had no significant causal impact on these immune phenotypes, suggesting that the occurrence of osteomyelitis may not affect these immune cell phenotypes.

**Conclusion:**

To our knowledge, this is the first study to shed light on the causal relationship between specific immune cell characteristics and the development of osteomyelitis, thereby providing a new perspective to understand the immune mechanism of osteomyelitis. These findings are significant for formulating targeted prevention and treatment strategies, and hold promise to improve the treatment outcomes for patients with osteomyelitis.

## 1 Introduction

Osteomyelitis represents one of the most severe and treatment-resistant bone infections, characterized by inflammatory destruction of the bone and bone marrow tissues driven by aggressive and dysregulated host immune responses (Lew and Waldvogel, 2004). Both acute and chronic forms of osteomyelitis remain major challenges in musculoskeletal medicine and impose substantial clinical and economic burden worldwide (Kremers et al., 2015b). In the United States alone, the annual incidence of osteomyelitis is approximately 1 in 5,000 children, while in low- and middle-income countries, limited diagnostic facilities and antibiotic access further exacerbate osteomyelitis-associated disability and morbidity (Hatzenbuehler and Pulling, 2011; Kremers et al., 2015a). Despite therapeutic advancements, successful treatment of osteomyelitis continues to be thwarted by the ability of pathogens to evade host defenses and antibiotics by forming resilient biofilms within the avascular sequestrum areas of necrotic bone, coupled with the emergence of multidrug-resistant strains (Costerton et al., 1999). Therefore, better understanding of the intricate immunopathogenic mechanisms governing osteomyelitis development and progression is imperative to enable the identification of novel preventive, prognostic, diagnostic, and therapeutic strategies.

A major determinant of osteomyelitis outcomes is the nature of the host innate and adaptive immune responses against invading pathogens. While controlled inflammation is crucial for microbial clearance and bone repair, excessive or improperly regulated inflammation can potentiate irreversible bone destruction and damage (Mader et al., 1999). For instance, hyper-stimulation of osteoclasts by pro-inflammatory cytokines such as interleukin-1 (IL-1), interleukin-6 (IL-6), and tumor necrosis factor-α (TNF-α) can prompt aggressive and permanent bone resorption (Teitelbaum, 2000). Dysregulated inflammation can also disrupt coupling between bone formation and resorption, hence impairing bone remodeling and repair (Sims and Martin, 2014). Furthermore, the formation of rigid sequestrum areas consisting of avascular and necrotic bone in chronic osteomyelitis creates an immunologically privileged niche that facilitates the persistence of pathogens, sustaining chronic inflammation and progressive bone loss (Lazzarini et al., 2004). Hence, clarifying how specific perturbations in immune cell phenotypes causally influence osteomyelitis susceptibility and severity is paramount, as this knowledge can pave the way for targeted immunomodulatory therapies that can resolve deleterious inflammation while retaining the protective antimicrobial immunity.

The current understanding of immune responses in osteomyelitis is derived predominantly from animal models and observational analyses of patient samples (Gómez-Barrena et al., 2015; Birt et al., 2017). While these approaches have offered clues in terms of the participating immune components such as neutrophils, macrophages, T cells, and B cells, they have limitations that impede the definitive establishment of causal relationships between immune traits and osteomyelitis risk. Confounding factors, reverse causation, and the inability to arrive at interventional inferences are key restrictions of conventional observational studies that necessitate alternative approaches (Davey Smith and Hemani, 2014). Mendelian randomization (MR) is one such powerful technique that leverages genetic variants as instrumental variables (IVs) to strengthen causal inferences and overcome biases inherent in observational analyses (Hemani et al., 2018; Little, 2018). MR provides a framework to delineate causal effects of modifiable exposures on disease outcomes by minimizing issues such as confounding factors that affect traditional regression analyses (Lawlor et al., 2008).

Herein, we performed a comprehensive MR study to systematically evaluate the causal associations between a broad range of immune cell phenotypes, encompassing innate and adaptive populations, and genetic susceptibility to osteomyelitis. By clarifying the causal roles of specific immune cell subsets and activation states in osteomyelitis pathogenesis, this study aimed to advance the understanding of the immunological landscape governing this refractory bone infection and uncover potential prognostic biomarkers and therapeutic targets to improve prevention, monitoring, and clinical management of osteomyelitis. Elucidating causal immune factors influencing osteomyelitis risk can provide significant insights into disease mechanisms and guide the development of targeted immunomodulatory interventions that resolve detrimental inflammation while retaining protective antimicrobial immunity.

## 2 Materials and methods

### 2.1 Study design

Based on MR analyses of the two samples, we assessed the causal associations between 731 immune cell traits (seven groups: B cells, cDCs, myeloid cells, mature T cells, monocyte, TBNK and Treg cells) and osteomyelitis. MR uses genetic variation to represent risk factors, and therefore valid IVs in causal inference must satisfy three key assumptions (Lew and Waldvogel, 2004): genetic variation is directly associated with exposure (Kremers et al., 2015b); genetic variation is not associated with confounders between exposure and outcome; and (Hatzenbuehler and Pulling, 2011) genetic variation will not influence outcome through pathways other than exposure (Yang et al., 2023). Data on osteomyelitis were obtained from the FinnGen R5, which included 210,417 Europeans that were genotyped for GWAS. Among these Europeans, 842 had osteomyelitis and served as GWAS cases, while the remaining 209,575 were controls. The GWAS comprised 16,380,449 single nucleotide polymorphisms (SNPs) (Dai et al., 2023).

### 2.2 Immunity-wide GWAS data sources

The total GWAS statistics for each immunological profile are publicly available from the GWAS catalog (registry numbers GCST90001391 to GCST90002121). A total of 731 immunophenotypes were included, including absolute cell counts (AC) (n = 118), median fluorescence intensity (MFI) reflecting surface antigen levels (n = 389), morphology parameter (MP) (n = 32), and relative cell count (RC) (n = 192). Specifically, the MFI, AC, and RC features contained B cells, conventional dendritic cells (cDCs), mature T cells, monocytes, myeloid cells, TBNK (T cells, B cells, natural killer cells) and regulatory T (Treg) cells, while the MP feature contained cDCs and TBNK cells only (Kanazawa, 2022). The original immune signature GWAS used data from 3,757 Europeans with no overlap. SNPs were calculated for approximately 22 million high-density array genotypes using a reference panel based on Sardinian sequences, and correlations were examined after adjusting for covariates (i.e., sex and age) (Cheng et al., 2020).

### 2.3 Selection of IVs

The significance level of IVs for each immune characteristic was set at 5 × 10^−6^, because genetic variation is directly related to exposure (Labrecque and Swanson, 2021). To obtain site-independent IVs, we used the “TwoSampleMR” packet data with a linkage disequilibrium (LD) threshold set at R^2^ < 0.001 and an aggregation distance of 10,000 kb (Hughes et al., 2014). For osteomyelitis, we adjusted the significance level to 1 × 10^−5^, which is typically used to indicate genome-wide significance in GWAS, with an LD threshold of R^2^ < 0.001 and an aggregation distance of 10,000 kb.

### 2.4 Statistical analysis

**FIGURE 1:**
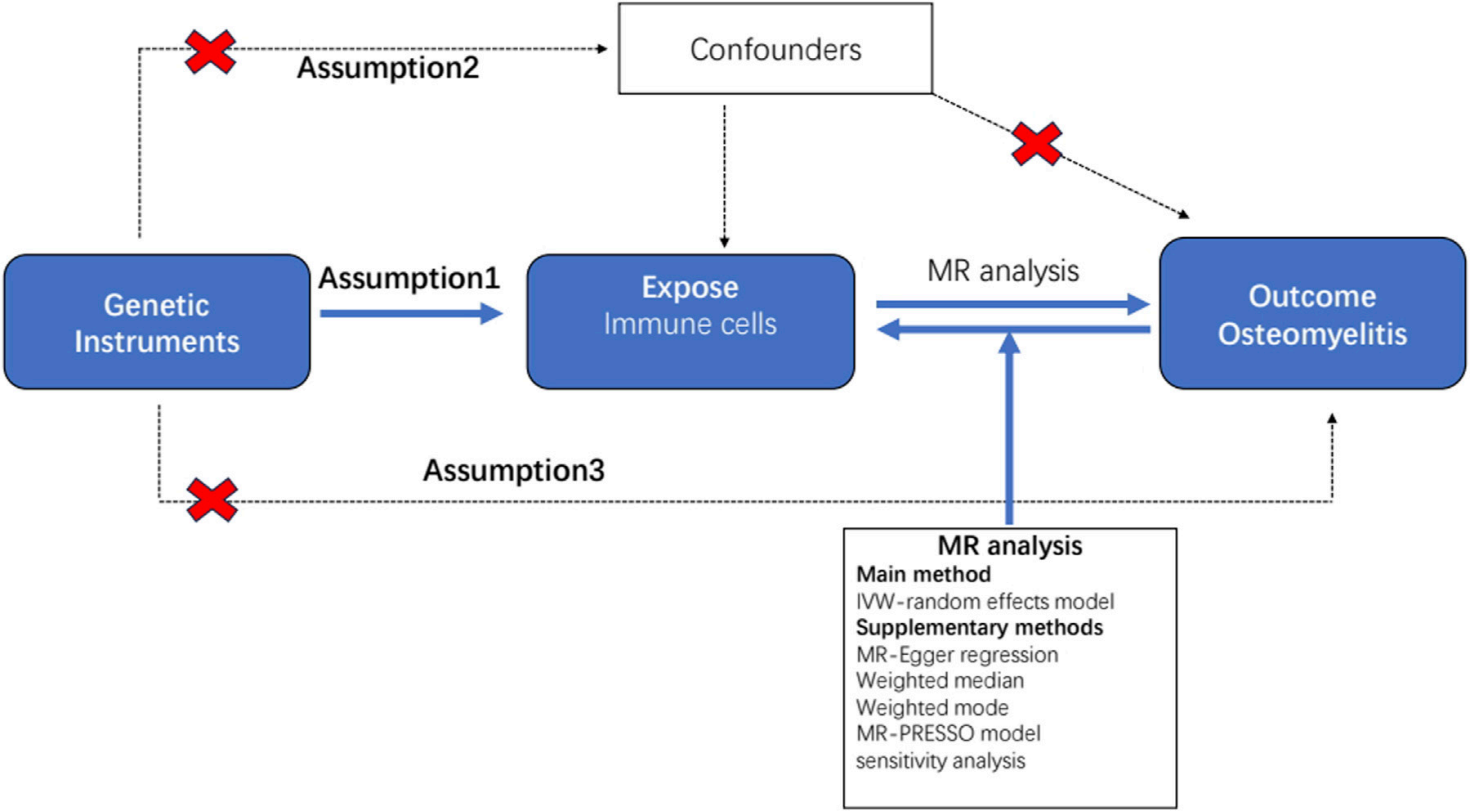
Flow diagram for quality control of the instrumental variables (IVs) and the entire Mendelian randomization (MR) analysis process. Abbreviations: SNPs, single-nucleotide polymorphisms; IVW, inverse variance weighted; MR, Mendelian randomization; MR Presso, Mendelian Randomization Pleiotropy RESidual Sum and Outlier.

All statistical analyses were performed using the R software (version 4.2.1) (http://www.Rproject.org) (Daly and Soobiah, 2022). To determine the causal relationship between the 731 immunophenotypes and osteomyelitis, we mainly used inverse variance weighting (IVW) and weighted median (Xu et al., 2022). These analyses were carried out by the “TwoSampleMR” package (version 0.5.7) in the R software environment (Yang et al., 2021). This package is specifically designed for performing MR analyses and provides tools for estimating, testing, and sensitivity analyses of causal effects. The IVW method is a standard method in MR that combines Wald analysis (ratio of SNP outcome associations to SNP exposure associations) from multiple genetic variants, weighted by the inverse variance of each SNP outcome association (Bowden et al., 2016). Weighted median and model-based approaches were used as complementary methods to provide reliable causal estimates even when some IVs were invalid, provided certain assumptions were met (Hartwig et al., 2017). These analyses are supported by rigorous sensitivity analysis, including Cochran’s Q-test, to test for heterogeneity between instrumental variables. We performed a stability selection analysis using the leave-one-out method to complement our sensitivity analyses (Bowden et al., 2017). This thorough statistical assessment ensured that findings regarding the relationship between immunophenotypes and osteomyelitis were as reliable and accurate as possible. A detailed flowchart of the analysis is shown in Figure 1.

## 3 Results

### 3.1 Exploration of the causal effect of immunophenotypes on osteomyelitis risk

**FIGURE 2:**
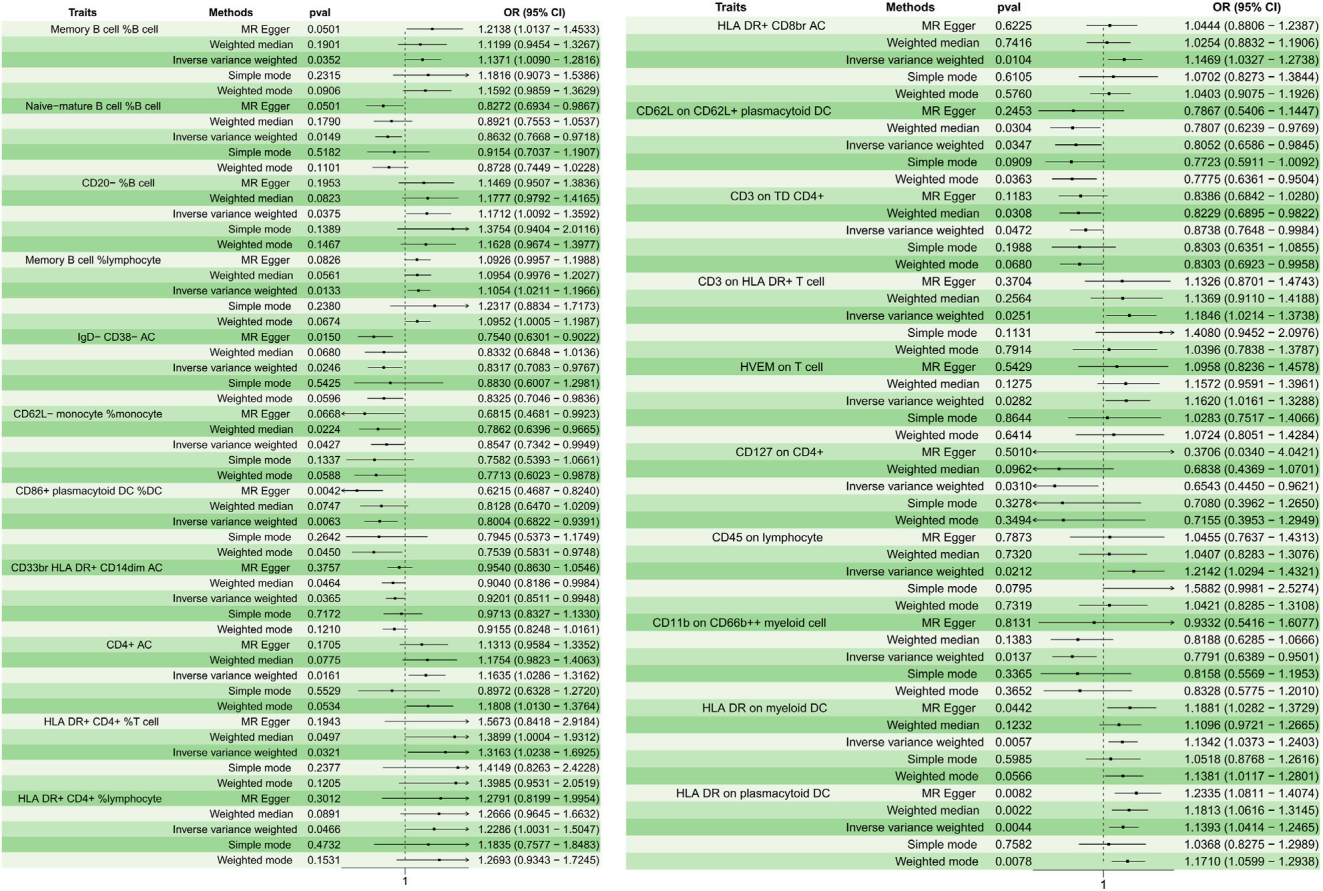
Forest plots depicting the causal associations between osteomyelitis and specific immune cell traits. Abbreviations: IVW, inverse variance weighting; CI, confidence interval; HVEM, Herpesvirus-entry mediator; DC, dendritic cell; HLA, human leukocyte antigen; TD, delayed reaction T cell; CD, cluster of differentiation.

A total of 21 immunophenotypes were identified to be causally associated with the development of osteomyelitis at a significance level of 0.05. The genetic instruments for each immune phenotype can be found in Supplementary Table S1. There were five cases in the B-cell group, five in the cDCs cell group, three in the Myeloid cell group, five in the TBNK cell group, two in the Maturation stages of T cell group, and one in the Treg cell group (Figure 2).

In the B cell group, Memory B cell %B cell (*p* = 0.035, OR = 1.137, 95%CI = 1.008–1.281); CD20^−^ %B cell (*p* = 0.037, OR = 1.171, 95%CI = 1.009–1.359); and Memory B cell % lymphocyte (*p* = 0.013, OR = 1.105, 95%CI = 1.021–1.196) showed a positive causal relationship with the development of osteomyelitis, while Naive-mature B cell %B cell (*p* = 0.014, OR = 0.863, 95%CI = 0.776–0.971) and IgD- CD38^−^ AC (*p* = 0.024, OR = 0.831, 95%CI = 0.708–0.976) showed a negative causal relationship with the development of osteomyelitis. In cDC cells, HLA DR on myeloid DC (*p* = 0.005, OR = 1.134, 95%CI = 1.037–1.240) and HLA DR on plasmacytoid DC (*p* = 0.004, OR = 1.139, 95%CI = 1.041–1.246) showed a positive causal relationship, while CD62L on CD62L + plasmacytoid DC (*p* = 0.034, OR = 0.805, 95%CI = 0.658–0.984); CD62L-monocyte %monocyte (*p* = 0.042, OR = 0.854, 95%CI = 0.734–0.994); and CD86^+^ plasmacytoid DC %DC (*p* = 0.006, OR = 0.800, 95%CI = 0.682–0.939) showed a negative causal relationship with the development of osteomyelitis. In the Myeloid cell group, CD45 on lymphocyte (*p* = 0.021, OR = 1.214, 95%CI = 1.029–1.432) showed a positive causal relationship with the development of osteomyelitis, while CD33br HLA DR + CD14dim AC (*p* = 0.036, OR = 0.920, 95%CI = 0.851–0.994) and CD11b on CD66b++ myeloid cell (*p* = 0.013, OR = 0.779, 95%CI = 0.638–0.950) showed a negative causal relationship with the development of osteomyelitis. In the TBNK cell group, all immune features showed a positive causal relationship with the development of osteomyelitis: CD4^+^ AC (*p* = 0.016, OR = 1.163, 95%CI = 1.028–1.316); HLA DR+ CD4^+^ %T cell (*p* = 0.032, OR = 1.316, 95%CI = 1.023–1.692); HLA DR+ CD4^+^ %lymphocyte (*p* = 0.046, OR = 1.228, 95%CI = 1.003–1.504); HLA DR + CD8br AC (*p* = 0.010, OR = 1.146, 95%CI = 1.032–1.273); and CD3 on HLA DR + T cell (*p* = 0.025, OR = 1.184, 95%CI = 1.021–1.373).

In the maturation stages of the T cell group, herpesvirus-entry mediator (HVEM) on T cell (*p* = 0.028, OR = 1.161, 95%CI = 1.016–1.328) showed a positive causal relationship with the development of osteomyelitis, and CD3 on delayed reaction T cell (TD) CD4^+^ (*p* = 0.047, OR = 0.873, 95%CI = 0.764–0.998) showed a negative causal relationship with the development of osteomyelitis. In the Treg cell group, CD127 on CD4^+^ (*p* = 0.031, OR = 0.654, 95%CI = 0.445–0.962) showed a negative causal relationship with the development of osteomyelitis. The results of sensitivity analyses showed the robustness of the observed causal associations (Supplementary Figure S1). Scatterplots and funnel plots also showed the stability of the results (Supplementary Figures S2, S3).

### 3.2 Exploration of the causal effect of osteomyelitis risk on immunophenotypes

To investigate the causal relationship between osteomyelitis and immune phenotype, two-sample MR analysis was used, with the IVW method as the primary analysis method and other methods as auxiliary methods. Then, we used inverse MR to study the effect of osteomyelitis on immune phenotype cells. The results showed that there was no causal relationship between osteomyelitis and any of the above-mentioned 21 immune cells.

## 4 Discussion

This two-sample MR study provides novel evidence for causal associations between specific immune cell phenotypes and susceptibility to osteomyelitis. The findings highlight 21 immune cell traits across B cells, DCs, myeloid cells, TBNK cells, T cell maturation stages, and Treg cells that demonstrate significant causal relationships with osteomyelitis risk. Our results have important implications for furthering our understanding of osteomyelitis pathogenesis, identifying potential biomarkers, and developing targeted immunomodulatory treatments.

Overall, the study reveals a complex interplay between both pro- and anti-inflammatory immune phenotypes in influencing osteomyelitis development. Increased proportions of memory B cells and decreased naive B cells suggest that adaptive immune memory may enhance susceptibility to osteomyelitis. This aligns with the evidence that memory B cells mediate more rapid and heightened antibody responses upon re-exposure to pathogens (Tangye et al., 2015; Cyster and Allen, 2019). Conversely, depletion of naive B cells, which typically demonstrate more restricted specificity, may hamper initial pathogen control. The positive association of memory B cell proportions with osteomyelitis risk emphasizes the likely role of humoral immunity in driving excessive inflammation during infection.

Furthermore, reduced IgD-CD38-naive B cells linked to lower osteomyelitis risk also indicates that impaired early B cell responses could ameliorate disease severity. As IgD + CD38^−^ B cells represent a subpopulation of mature naive B cells primed for activation (Nutt et al., 2015), their decline may attenuate acute inflammatory responses to osteomyelitis-causing pathogens. This aligns with observations in other inflammatory conditions like lupus, where lower IgD + CD38^−^ B cells are associated with reduced disease activity (Sanz et al., 2008). The contrasting effects of memory and naive B cell phenotypes highlight the delicate balance between efficient pathogen clearance and uncontrolled inflammation that likely shapes osteomyelitis outcomes.

The identification of HLA-DR expression on DCs as an osteomyelitis risk factor also provides further insights into antigen presentation processes that may drive inflammation in osteomyelitis. HLA-DR enables dendritic cells to activate T cells by presenting pathogen-derived peptides, thus initiating adaptive responses (Steinman and Banchereau, 2007). Elevated HLA-DR levels on myeloid and plasmacytoid DCs may therefore lead to excessive T cell stimulation, perpetuating inflammatory damage characteristic of osteomyelitis. This finding reveals new facets of DC involvement in skewing T cell responses during bony infection, warranting further characterization.

Conversely, reduced frequencies of CD62L-expressing monocytes and CD86^+^ plasmacytoid DCs linked to lower osteomyelitis risk suggest that restrained antigen presentation capacity in these populations may mitigate inflammation. Loss of CD62L and CD86, molecules crucial for antigen presentation and T cell co-stimulation (Lanzavecchia and Sallusto, 2001), could attenuate dendritic cell activation of T cells. This highlights a possible mechanism by which downregulation of DC stimulatory capacity may protect against exacerbated osteomyelitis inflammation.

The observed effects of myeloid cell phenotypes offer additional insights into innate immune processes influencing osteomyelitis pathogenesis. Elevated CD45, a tyrosine phosphatase regulator of immune cell activation (Hermiston et al., 2003), on lymphocytes increased osteomyelitis risk. This implies that enhancing early lymphocyte responsiveness to infection may worsen osteomyelitis outcomes. By contrast, reduced frequencies of CD33 + HLA-DR + monocytes and CD11b expression on myeloid cells linked to lower osteomyelitis risk suggest that constraining the activation and adhesive capacity of these innate populations could alleviate damage due to inflammation. CD33 and CD11b enable myeloid cell recruitment and pathogen response (van der Touw et al., 2017); hence, their downregulation may limit detrimental effects of myeloid hyperactivity. Together, these results highlight how altering lymphocyte and myeloid cell responsiveness could shape osteomyelitis susceptibility.

The TBNK phenotypes demonstrating positive associations with osteomyelitis risk provide clues into how augmenting T and NK cell inflammatory activity could enable detrimental immune-mediated bone damage. Elevations in circulating CD4^+^ T cells, potentially enriching Th1 and Th17 proinflammatory subsets, as well as increases in HLA-DR+ CD4^+^ and CD8^+^ T cells indicative of activation were linked to higher osteomyelitis risk. This implies that increased accumulation and stimulation of pathogen-responsive T cell subsets may drive osteomyelitis immunopathology. The contribution of CD3 signaling strength in activated T cells further substantiates the involvement of T cell hyper-responsiveness in osteomyelitis pathogenesis. The positive correlation between activated NK cell levels and osteomyelitis risk also accords with evidence that NK cells can induce bone loss through stimulating osteoclast differentiation and activity (Söderström et al., 2010). Together, these findings reveal that T and NK cell phenotypes are likely to ignite damaging inflammation in osteomyelitis.

The observed effects of T cell maturation patterns provide additional insights into how tuning T cell inflammatory capacity could impact osteomyelitis outcomes. Greater expression of HVEM, which can act as a T cell co-stimulator (Cheung et al., 2009), associated with increased osteomyelitis risk highlights that enhancing T cell activation may exacerbate bone inflammation. Conversely, reduced CD3 signaling strength in TD CD4^+^ T cells linked to protection against osteomyelitis suggests that dampening naïve T cell responsiveness could alleviate inflammation. This indicates that modulating T cell maturation and activation status could crucially shape osteomyelitis susceptibility.

The negative causal association between Treg frequencies and osteomyelitis risk comes along with a protective role for immunosuppressive Tregs in restraining inflammatory bone damage. Tregs expressing CD127 enable immune tolerance (Sakaguchi et al., 2008), thus their decline may remove a key obstacle in osteomyelitis inflammation. This is consistent with findings that Treg supplementation alleviates osteomyelitis in animal models (Zaiss et al., 2007), confirming their therapeutic potential. Overall, the collective insights into T cell phenotypes emphasizes the likely involvement of both amplifying T effector responses and impairing Treg-mediated immune regulation in driving osteomyelitis pathogenesis.

The application of MR in this study enables these causal immune associations to be identified while minimizing the limitations of conventional observational research. The use of genetic variants as IVs circumvents biases from confounding variables and reverse causation that typically restrict observational analyses (Davey Smith and Hemani, 2014). This facilitates more robust causal inference, allowing for the delineation of immune traits that influence osteomyelitis risk. The bi-directional analysis also revealed that osteomyelitis *per se* did not causally impact the identified immune phenotypes. This implies that while certain immune cell features may drive osteomyelitis development, the disease itself does not physically alter these specific parameters.

Our findings carry valuable implications for translating insights on osteomyelitis pathogenesis into clinical practice. The immune phenotypes causally linked to osteomyelitis risk could be evaluated as potential prognostic biomarkers to enable early risk stratification and guide clinical monitoring in patients. Memory B cells, DC HLA-DR expression, and frequencies of activated T cell subsets may hold promise as predictors of osteomyelitis development or severity (Kawai et al., 2017). Additionally, the identified immune cell traits could represent novel therapeutic targets for immunomodulatory treatments that control detrimental inflammation while retaining the protective responses. Selective depletion of memory B cells, augmentation of naive B cells, and timed Treg supplementation represent possible avenues for exploration.

This study has some limitations. First, the MR analyses used data predominantly from European cohorts, necessitating confirmation in other populations. Integrating detailed clinical metadata could also help assess the effects of factors such as age, sex, and comorbidities (Little, 2018). Second, the specific molecular mechanisms by which the identified cell types influence osteomyelitis pathogenesis warrant further characterization through *in vitro* and *in vivo* investigations. How shifts in these immune cell phenotypes specifically impact processes like leukocyte migration, microbial clearance, bone remodeling, and inflammatory signaling require deeper mechanistic interrogation. Third, as genetic instruments only capture the variations during the life cycle of cells and their subsets, changes in their levels arising during active osteomyelitis could not be addressed. Longitudinal tracking of fluctuations in these immune traits pre- and post-osteomyelitis onset could provide further temporal insights. Exploring interactions between immune phenotypes in relation to osteomyelitis using mediation analyses may also clarify interdependencies between leukocyte subsets (Hemani et al., 2018). Additionally, the inherent limitations of MR approaches should be acknowledged, including potential pleiotropic effects of genetic variants and limited power when using modest sample sizes. Fourth, While our analysis provides robust evidence using genetic variants as instrumental variables, the inherent limitations of observational data and the potential for unmeasured confounding factors highlight the need for subsequent validation in independent cohorts. Specifically, experimental studies in diverse populations and different environmental contexts are crucial to establish the direct impact of these immune cell phenotypes on osteomyelitis development and progression. This step is vital for translating our findings from genetic associations into clinically relevant interventions and understanding the complex immunological mechanisms underlying osteomyelitis. Therefore, we advocate for the replication of our results through experimental approaches to confirm causality and inform the development of targeted therapies.

However, it is important to note that bottleneck populations and island populations, due to their unique genetic structures and historical demographic events, may affect the accuracy and generalizability of MR analysis. Firstly, the genetic variability in bottleneck and island populations might be limited due to historical reductions in population size and geographical isolation. This specificity in genetic structure could potentially limit our MR analysis to specific population backgrounds, affecting the universal applicability of our results. Nonetheless, by selecting strong IVs directly associated with immune cell characteristics and conducting sensitivity analyses, we have endeavored to minimize the impact of this issue. Secondly, the genetic heterogeneity of bottleneck and island populations could lead to a unique correlation between genetic instrumental variables and environmental factors, possibly violating the no confounding assumption in MR analysis. We have assessed this potential bias using multiple complementary methods, such as the weighted median method, to ensure the robustness of our findings.

In summary, despite the aforementioned limitations, our study provides new insights into the causal relationship between immune cell characteristics and the development of osteomyelitis. Future research should consider the diversity of population genetic structures to further deepen the understanding of the immunological mechanisms of osteomyelitis and provide a scientific basis for developing targeted prevention and treatment strategies.

## 5 Conclusion

In summary, this study provides compelling evidence that alterations in B cell, DC, myeloid cell, T cell, and NK cell phenotypes induce causal effects on osteomyelitis susceptibility. These findings substantially advance understanding of the immunological landscape underlying osteomyelitis pathogenesis. This knowledge could pave the way for biomarker development, patient stratification, and targeted immunomodulatory interventions to improve the prevention and management of this debilitating bone infection. To strengthen the evidence base and ensure the generalizability of our conclusions, we advocate for the replication of our findings across different populations and with additional genetic datasets. Such replication efforts are warranted to validate the causal associations we have identified and to account for potential population stratification, environmental influences, and genetic heterogeneity that may affect the observed relationships. Further research on these observations holds promise for translating new insights on osteomyelitis immunopathology into tangible clinical benefits.

## Data Availability

The original contributions presented in the study are included in the article/Supplementary Material, further inquiries can be directed to the corresponding author.
