# Supplementary material for: Exploring the immunological landscape of osteomyelitis through mendelian randomization analysis
Source: Front Genet. 2024 Apr 8;15:1362432. doi: 10.3389/fgene.2024.1362432 (PMC11033344; doi:10.3389/fgene.2024.1362432)
Supplement: Supplementary file 1 [file Table2.DOCX]

Supplementary Material

# Supplementary Figures 1


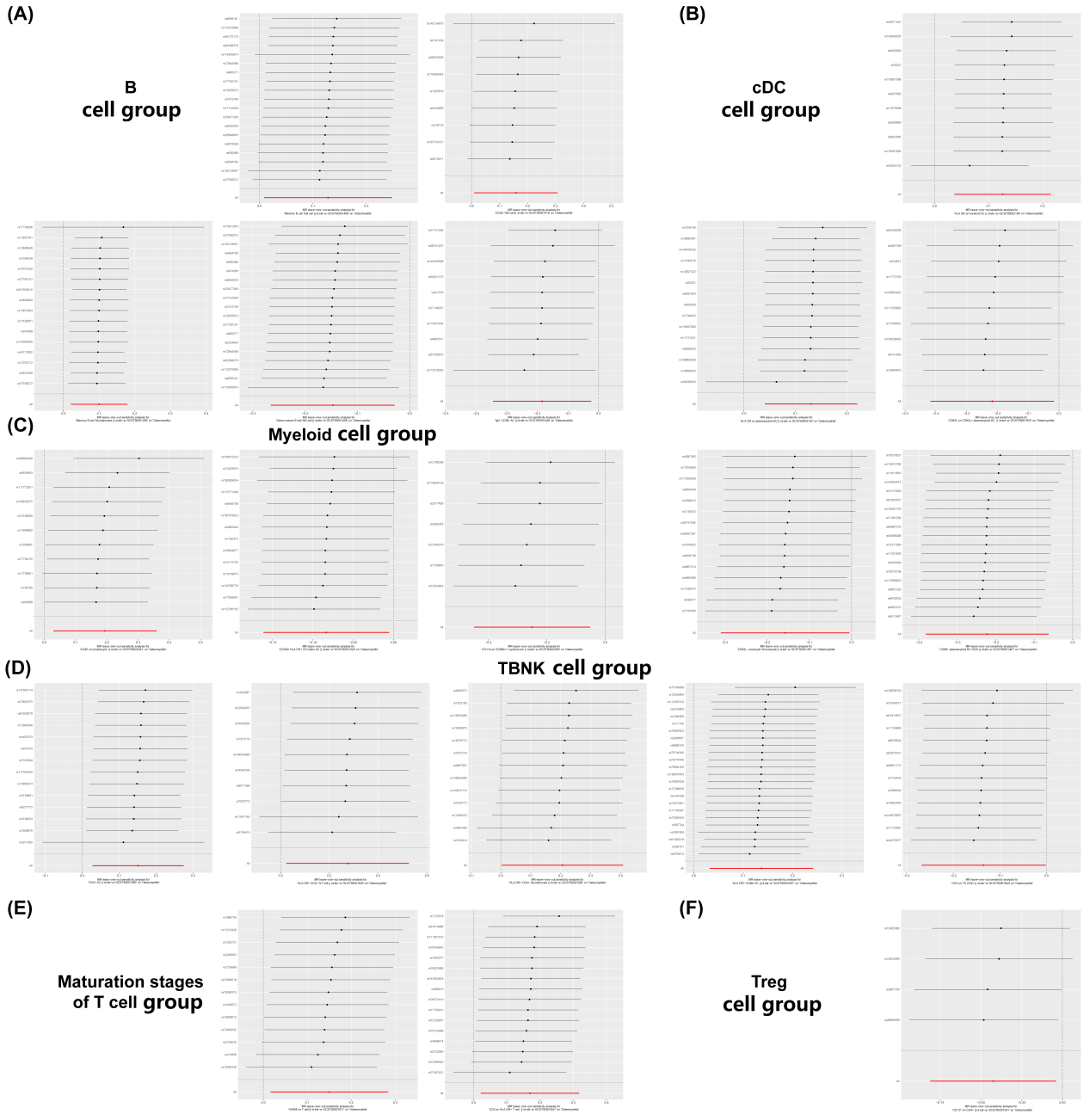


**Supplementary Figure 1.** Each row was deemed as an independent MR analysis for estimating the immune cells and osteomyelitis causal effect using all of the remaining IVs except for the single SNP listed on the y-axis. And where all dots locate were required to be greater than zero on the x-axis. (A)B cell group sensitivity analyses plot (B)cDC cell group sensitivity analyses plot (C)Myeloid cell group sensitivity analyses plot (D)TBNK cell group sensitivity analyses plot (E)Maturation stages of cell group sensitivity analyses plot (F)Treg cell group sensitivity analyses plot

# Supplementary Figures 2


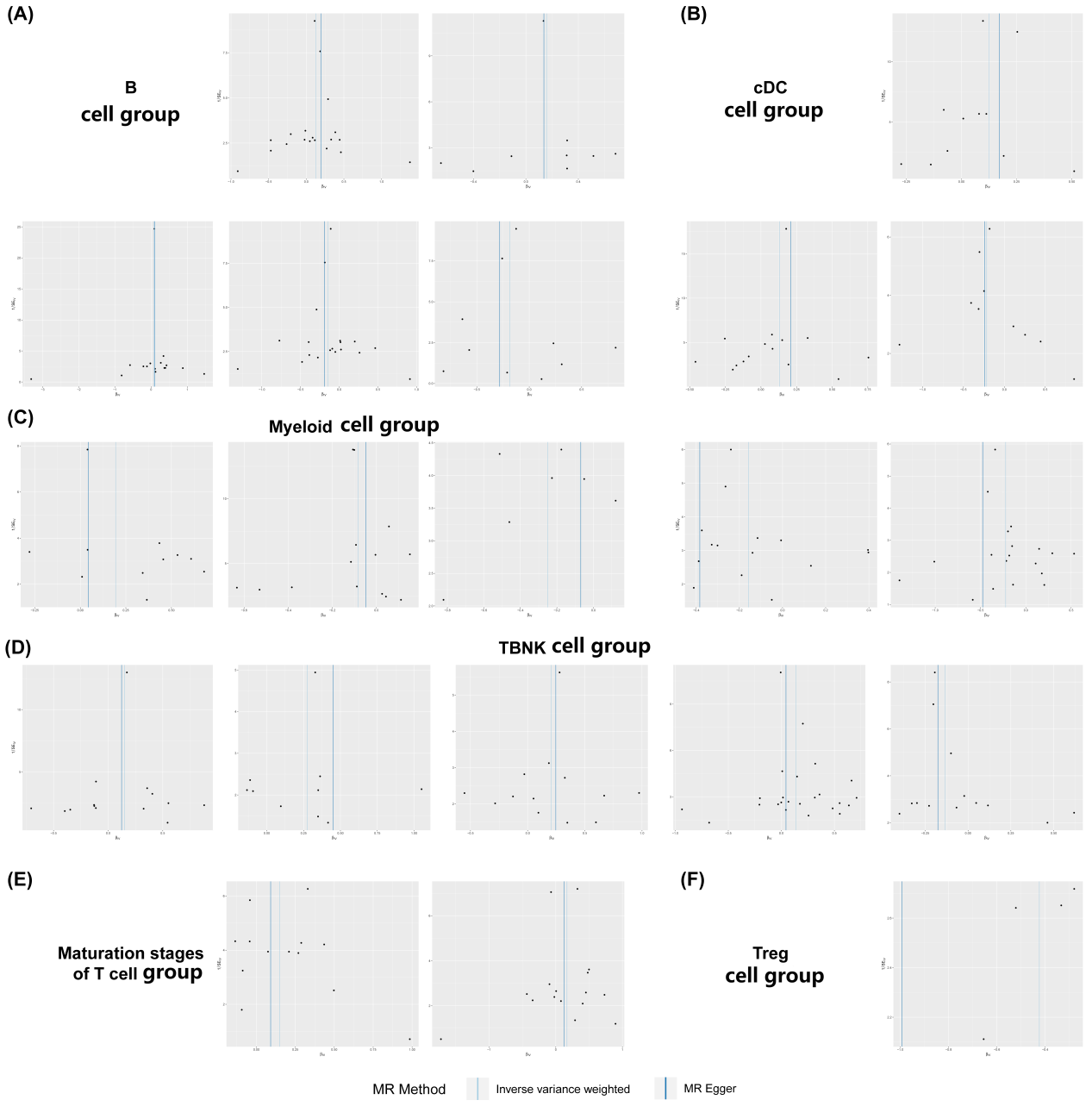


**Supplementary Figure 2.** Funnel plot to evaluate the robustness. The scattered points indicated the estimated effect of a single SNP used as an instrumental variable. The vertical lines represented the global estimate derived from the inverse variance weighted method and MR-Egger regression. (A)B cell group sensitivity funnel plot (B)cDC cell group funnel plot (C)Myeloid cell group funnel plot (D)TBNK cell group funnel plot (E)Maturation stages of cell group funnel plot (F)Treg cell group funnel plot

# Supplementary Figures 3


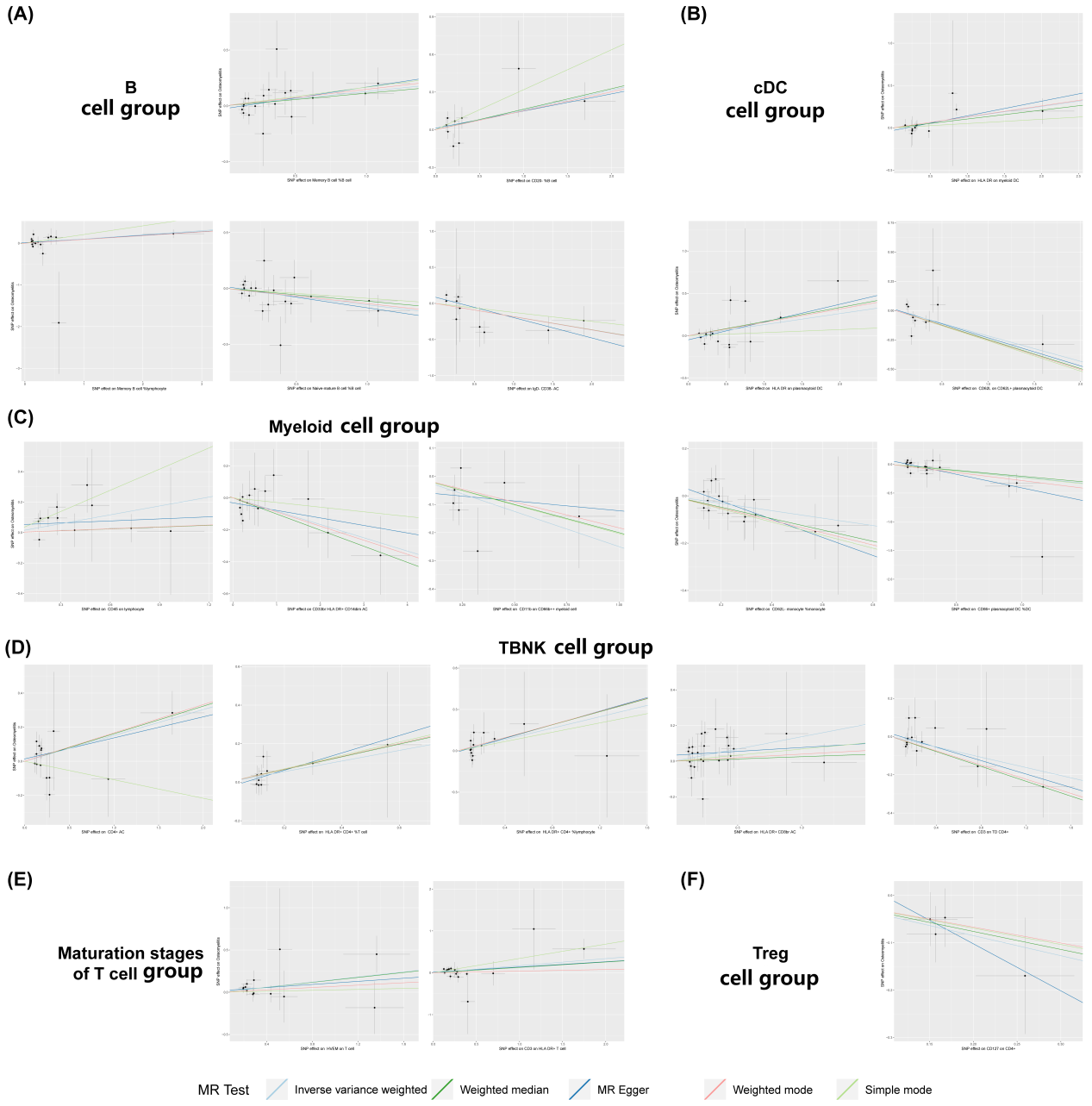


**Supplementary Figure 3.** Scatter plot of SNPs relevant to immune cells and the risk of osteomyelitis. Each splash displayed the effect sizes for SNP-immune cells relation (x-axis, SD units) and the SNP-osteomyelitis relation (y-axis, log (OR)) with 95% CIs. Using three MR strategies (the inverse variance weighted, MR-Egger, and weighted median) (R package “TwoSampleMR”), the regression slopes of the lines associated with the causal estimates were determined. (A)B cell group scatter plot (B)cDC cell group scatter plot (C)Myeloid cell group scatter plot (D)TBNK cell group scatter plot (E)Maturation stages of cell group scatter plot (F)Treg cell group scatter plot
